# Genome-Wide Association Study and Prediction of Tassel Weight of Tropical Maize Germplasm in Multi-Parent Population

**DOI:** 10.3390/ijms25031756

**Published:** 2024-02-01

**Authors:** Meichen Liu, Yudong Zhang, Ranjan K. Shaw, Xingjie Zhang, Jinfeng Li, Linzhuo Li, Shaoxiong Li, Muhammad Adnan, Fuyan Jiang, Yaqi Bi, Xingfu Yin, Xingming Fan

**Affiliations:** 1 School of Agriculture, Yunnan University, Kunming 650500, China; shirleyliu1028@163.com (M.L.); xingjiezhang2022@163.com (X.Z.); jinfengli1020@163.com (J.L.); lilinzhuo0606@163.com (L.L.); 15987701739@163.com (S.L.); 2 Institute of Food Crops, Yunnan Academy of Agricultural Sciences, Kunming 650205, China; mikezhangy@yahoo.com (Y.Z.); ranjanshaw@gmail.com (R.K.S.); alvi.adnan@yahoo.com (M.A.); jiangfuyansxx@126.com (F.J.); biyq122627@163.com (Y.B.); xingfuyin626@163.com (X.Y.)

**Keywords:** tassel weight, genome-wide association study, candidate gene, genomic selection

## Abstract

Tassel weight (TW) is a crucial agronomic trait that significantly affects pollen supply and grain yield development in maize breeding. To improve maize yield and develop new varieties, a comprehensive understanding of the genetic mechanisms underlying tassel weight is essential. In this study, tropical maize inbred lines, namely CML312, CML373, CML444, and YML46, were selected as female parents and crossed with the elite maize inbred line Ye107, which served as the common male parent, to develop a multi-parent population comprising four F8 recombinant inbred line (RIL) subpopulations. Using 6616 high-quality single nucleotide polymorphism (SNP) markers, we conducted genome-wide association analysis (GWAS) and genomic selection (GS) on 642 F8 RILs in four subpopulations across three different environments. Through GWAS, we identified 16 SNPs that were significantly associated with TW, encompassing two stable loci expressed across multiple environments. Furthermore, within the candidate regions of these SNPs, we discovered four novel candidate genes related to TW, namely *Zm00001d044362*, *Zm00001d011048*, *Zm00001d011049*, and *Zm00001d031173* distributed on chromosomes 1, 3, and 8, which have not been previously reported. These genes are involved in processes such as signal transduction, growth and development, protein splicing, and pollen development, all of which play crucial roles in inflorescence meristem development, directly affecting TW. The co-localized SNP, S8_137379725, on chromosome 8 was situated within a 16.569 kb long terminal repeat retrotransposon (LTR-RT), located 22.819 kb upstream and 26.428 kb downstream of the candidate genes (*Zm00001d011048* and *Zm00001d011049*). When comparing three distinct GS models, the BayesB model demonstrated the highest accuracy in predicting TW. This study establishes the theoretical foundation for future research into the genetic mechanisms underlying maize TW and the efficient breeding of high-yielding varieties with desired tassel weight through GS.

## 1. Introduction

Maize (*Zea mays* L.) is one of the most important cereal crops cultivated globally, and it plays a critical role in food, feed, and fuel production. Because of its monoecious characteristics, the tassel at the apex of the plant shades the lower leaves. This reduces light penetration, intensifies nutrient competition, decreases nutrient allocation to the female inflorescence, and affects the yield [1]. Pioneer Hi-Bred International Inc. in the USA examined maize hybrid varieties cultivated from 1967 to 1991, showing a notable 36% reduction in tassel weight during this period [2]. Previous research has also revealed that detasseling the same maize variety can significantly increase its yield [3]. Smaller tassels mitigate shading and nutrient competition with ears, especially in environments with high plant density, thereby fostering an increase in yield [4,5]. Therefore, the weight and structure of the tassel are significant determinants of maize yield [6]. Despite numerous studies confirming that larger and heavier maize tassels negatively influence yield, in practice, especially during hybrid seed production, breeders still prefer to select varieties with larger tassels as male parents to ensure a sufficient pollen supply [5,7]. With recent advancements in molecular markers and gene-mapping technologies, investigating TW and its regulatory mechanisms to efficiently breed new varieties with different tassel sizes and enhanced yield is of great significance.

GWAS has emerged as an essential tool for dissecting the molecular mechanisms of complex traits in both plants and animals. This analysis uses linkage disequilibrium and molecular markers covering the whole genome to scan functional gene loci associated with target traits, thereby enabling the identification of significant SNP loci with high precision. In maize, this technology has been extensively used to successfully detect genetic loci related to various traits, such as root structure [8], plant and ear height [9], kernel number per row [10], flowering time [11], and disease resistance [12,13]. Wu et al. utilized GWAS and linkage analysis to identify 125 quantitative trait loci (QTLs) and 965 quantitative trait nucleotides (QTNs) relevant to tassel branch number and length [14]. Qin et al. discovered and validated a QTN significantly associated with tassel branch number through an association mapping population and an F1 testcross population constructed using Mo17 [15]. In the context of TW, Berke and Rocheford (1999) utilized an F2 population comprising Illinois high-oil and low-oil maize varieties for QTL mapping of maize tassel branch number, branch angle, and weight, identifying seven QTLs related to TW [16]. Upadyayula et al. identified the genetic loci controlling TW on chromosomes 1, 3, 6, and 7 [5]. Wang et al. also detected 11 QTLs related to TW using two F2:3 populations derived by crossing Chinese elite maize inbred lines HuangZaoSi with Ye 478 and Qi 319, evaluated under six different environments [17]. Using different populations and methods, Liu et al. [18] and Xie et al. [19] also identified 10 and 3 QTLs, respectively, related to TW. Despite the identification of certain QTLs that regulate TW and related traits, the genetic mechanisms underlying this complex quantitative trait remain unclear. Notably, there has been relatively less research on genome-wide association analysis and candidate gene mining for TW.

Genome-wide selection utilizes high-density molecular markers covering the entire genome, such as SNPs, to estimate an individual’s genomic estimated breeding value (GEBV) or target traits and makes selections based on these breeding values [20]. Currently, this method has proven successful in numerous animal and crop breeding programs, facilitating the early prediction and selection of individuals, accelerating genetic gains, and effectively improving complex traits, while significantly shortening the generation interval and expediting the breeding process. In maize, GS has been used to improve various traits, including flowering time [21], general combining ability [22], plant height [23], kernel row number [24,25], disease resistance [26,27], and stalk strength [28]. However, there are few reports of genome-wide selection analyses of tassel structure and weight. Xu et al. utilized the ridge regression best linear unbiased prediction (rrBLUP) model for GS of tassel length, tassel primary branch number, tassel secondary branch number, and tassel branch number, with prediction accuracies of 0.36, 0.41, 0.28, and 0.38, respectively [29]. Dang et al. also employed the rrBLUP model for GS on tassel branch number in sweet corn and waxy corn, discovering that increasing the number of markers significantly associated with the target trait led to a notable improvement in prediction accuracy [30]. As the size of the training group increased from 10% to 90% of the total number of markers, the prediction accuracy for tassel branch number increased from 0.16 to approximately 0.48. Importantly, it is worth highlighting that studies on GS with TW as the target trait are yet to be conducted.

To explore candidate genes and their regulatory mechanisms associated with maize TW, we selected four elite inbred lines, namely CML312, CML373, CML444, and YML46 from the non-Reid and Suwan groups, along with Ye107 from the Reid group. By hybridizing these selected parental lines, we established a multi-parent population comprising 642 F8 RILs. This study employed a multi-parent population for GWAS and GS to identify the loci responsible for TW in maize. Our objectives encompassed (1) identifying significant SNPs associated with TW across different environments, (2) unveiling novel candidate genes that regulate TW via comprehensive functional annotation and GO enrichment analysis, and (3) assessing the predictive accuracy of TW under various GS models.

## 2. Results

### 2.1. Phenotypic Analysis of TW

A descriptive statistical analysis was conducted on tassel weight in a multi-parent maize population comprising four subpopulations across three different environments (Table 1). The results indicated that Pop1 exhibited the smallest mean TW in all environments, whereas Pop2 had the highest mean TW. The coefficient of variation for TW among the four subpopulations in each environment ranged from 0.31 to 0.39, confirming the differences among the samples. The absolute values of skewness and kurtosis for TW across different environments were both less than 1, indicating a normal distribution and consistent with the genetic characteristics of quantitative traits. Further analysis revealed that the heritability of each subgroup in different environments ranged from 33.00% to 33.40%, with lower heritability suggesting that this trait is highly influenced by environmental factors. Correlation analysis across different environments revealed significant positive correlations, such as between Yanshan in 2021 and Jinghong in 2022 (r = 0.872, *p* < 0.001). The phenotypic variation for TW was consistent across diverse environments, suggesting the reliability of these phenotypic data for subsequent analyses. Significant variations in TW among different subpopulations were observed and were found to be affected by environmental factors, laying a crucial foundation for further molecular mapping analysis.

### 2.2. Analysis of Population Structure in the Multi-Parent Population

**Figure 1:**
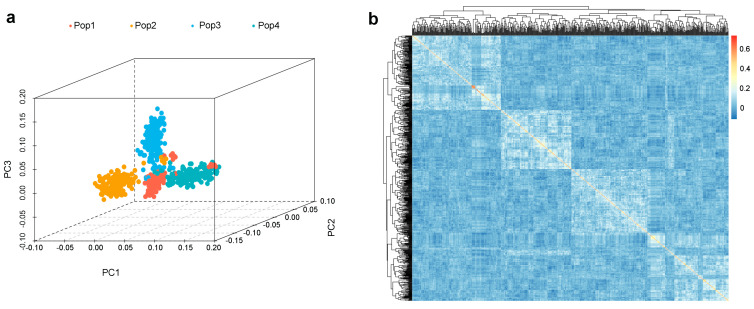
Multi-parent population structure analysis with 6616 SNPs. (**a**) Principal component analysis; red dots represent Pop1 (RIL-CML312), yellow dots represent Pop2 (RIL-CML373), blue dots represent Pop3 (RIL-CML444), and teal dots represent Pop4 (RIL-YML46). (**b**) Kinship analysis for 642 maize F8 RILs.

PCA revealed that these RILs could be divided into four distinct subpopulations (Figure 1a). The admixture observed among the subpopulations may be due to gene introgression or hybridization during breeding. The kinship relationship further revealed four significant subgroup structures (Figure 1b); however, these subpopulations could be classified into three major groups. This finding is consistent with the previously published theory of a three-heterotic-group pattern [31]. Among these, Pop1 (RIL-CML312), Pop2 (RIL-CML373), and Pop3 (RIL-CML444) belong to the non-Reid heterotic group, while Pop4 (RIL-YML46) belongs to the Suwan heterotic group.

### 2.3. Genome-Wide Association Analysis of TW in Multi-Parent Population

**Figure 2:**
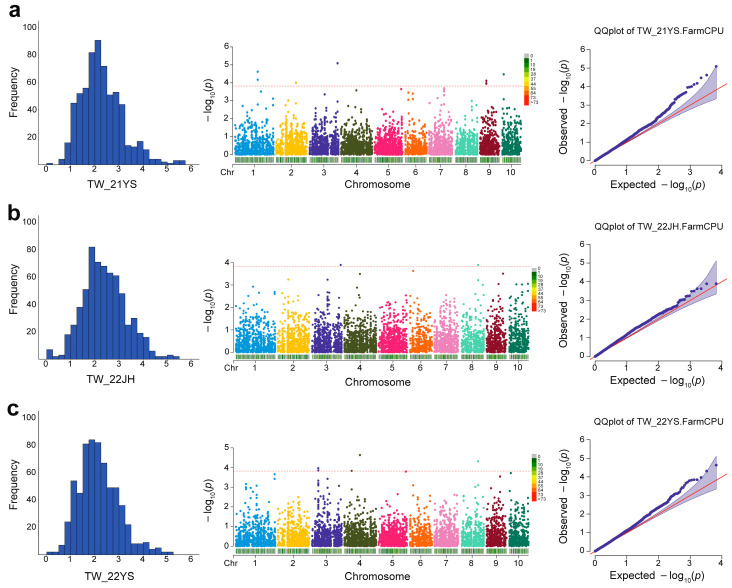
Phenotypic distribution, Manhattan, and QQ plots of genome-wide association study for TW in Yanshan in 2021 (**a**), Jinghong in 2022 (**b**), and Yanshan in 2022 (**c**). Manhattan plot: the dashed red line indicates the significance level. QQ plot: the red line indicates the expected significance value.

In this study, the TW of the multi-parent population in all three environments followed a normal distribution, which can be used for GWAS (Figure 2). Based on the phenotypes of 642 F8 RILs and 6616 high-quality SNP markers, we employed the FarmCPU model to perform GWAS for TW across different environments. Setting the threshold at −log_10_*p* > 3.82, a total of 16 significant SNPs were detected (Table 2): 8 in Yanshan in 2021, 2 in Jinghong in 2022, and 6 in Yanshan in 2022. These SNPs were located on chromosomes 1, 2, 3, 4, 8, 9, and 10 (Figure 2). Notably, two significant SNPs, S3_226041015 and S8_137379725, located on chromosomes 3 and 8, respectively, were consistently detected across environments.

### 2.4. Genetic Effect Analysis of Co-Localized SNPs

**Figure 3:**
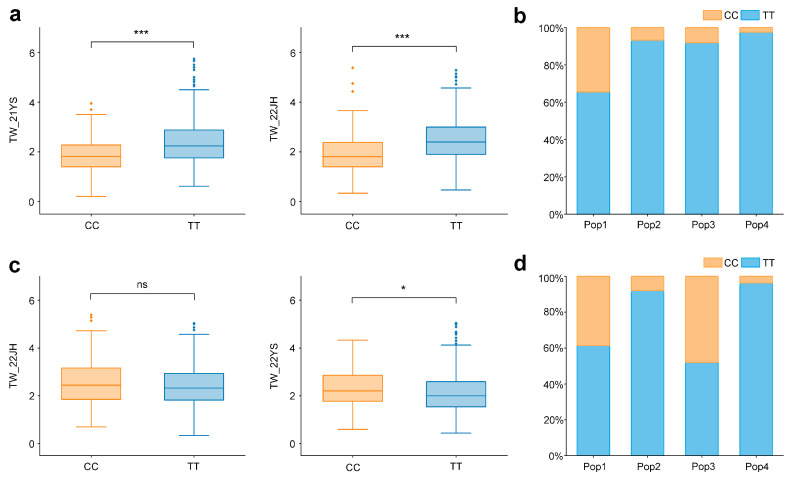
Phenotypic difference of different alleles of the co-localized SNPs. (**a**) Phenotypic difference of alleles at SNP S3_226041015 in the 21YS and 22JH environments, with *** indicating *p* < 0.001; (**b**) proportion of two alleles at SNP S3_226041015 observed in each subpopulation of the multi-parent population; (**c**) phenotypic difference of alleles at SNP S8_137379725 in the 22JH and 22YS environments, with ns indicating not significant and * indicating *p* < 0.05; (**d**) proportion of two alleles at SNP S8_137379725 observed in each subpopulation of the multi-parent population.

To gain a better and more precise understanding of the genetic effects of co-localized loci, this study conducted a comparative analysis of the allelic variation of the two co-localized SNPs associated with TW during GWAS (Figure 3). The results showed that at SNP S3_226041015, in the 21YS and 22JH environments, a C-T substitution occurred in the alleles. Maize plants with TT alleles exhibited significantly heavier TW than those with CC alleles (Figure 3a) (21YS: r = 0.170, *p* = 0.000016; 22JH: r = 0.176, *p* = 0.000008). Notably, among the four subpopulations in the multi-parent population, Pop1 showed the least allelic variation (Figure 3b), and when combined with the phenotype, this subpopulation also had the smallest mean TW. For SNP S3_226041015, the extent of the TT allele was positively correlated with TW. Similarly, in the 22JH and 22YS environments, another co-localized SNP, S8_137379725, also displayed an allelic transition from C to T. However, for this locus, maize RILs with TT alleles exhibited slightly lighter TW than those with the reference CC alleles (Figure 3c). Pop4 showed the highest allelic variation in the multi-parent population (Figure 3d) and had a lighter TW than the other subpopulations. At SNP S8_137379725, the TT allele was negatively correlated with TW. Overall, the variant alleles at both co-localized SNPs played a role in regulating maize tassel weight. Therefore, breeders can use variant alleles of these two SNPs to select TW in maize based on their specific breeding objectives. In the future, TW can be targeted for maize improvement through marker-assisted selection, facilitating more effective breeding of high-yielding varieties.

### 2.5. Functional Annotation and Enrichment Analysis of the Candidate Genes

**Figure 4:**
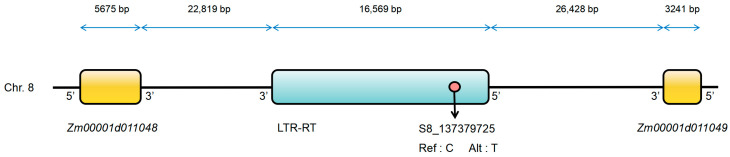
Relative positions of the significant SNP, LTR-RT, and candidate genes. Yellow boxes indicate candidate genes *Zm00001d011048* and *Zm00001d011049*. The blue box indicates the LTR-RT. The pink circle indicates the significant SNP, S8_137379725.

Through GWAS, 16 SNPs significantly associated with TW were identified. To further explore the candidate genes affecting TW, candidate genes located within 250 kb upstream and downstream of these significant SNPs were screened and annotated using databases, including MaizeGDB, InterPro, UniProt, and NCBI. In total, 17 genes were identified, of which 14 were functionally annotated or had homologs (Table 3). Notably, two SNPs, S3_226041015 and S8_137379725, which were consistently detected in different environments, had four genes identified within their candidate regions, namely *Zm00001d044359*, *Zm00001d044362*, *Zm00001d011048*, and *Zm00001d011049*. Three of these genes were functionally annotated or had homologous genes, except for *Zm00001d044359*. Furthermore, during candidate gene screening and annotation of the significant SNPs, it was found that the co-localized locus S8_137379725 is situated on a 16.569 kb long terminal repeat retrotransposon (LTR-RT). This LTR-RT was found to be located 22.819 kb upstream of the candidate gene *Zm00001d011048* and 26.428 kb downstream of the candidate gene *Zm00001d011049* (Figure 4).

**Figure 5:**
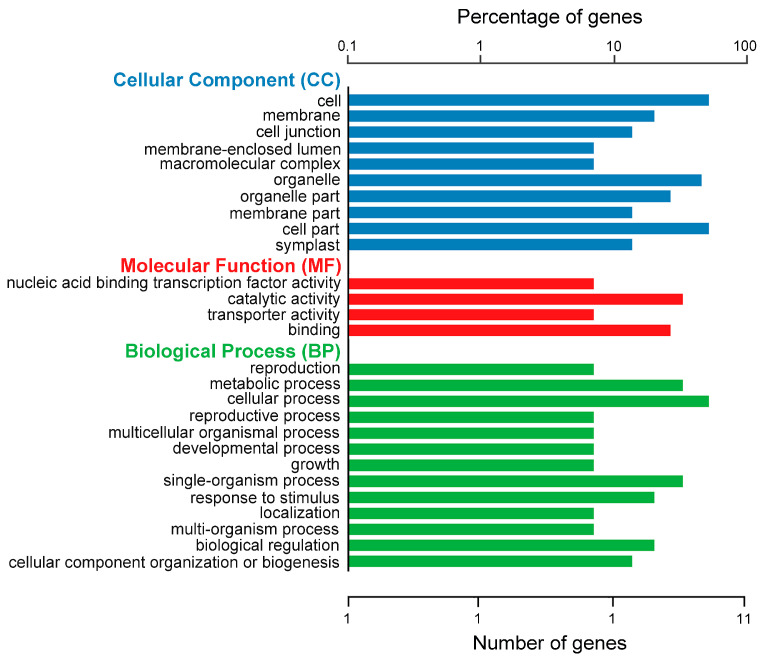
GO enrichment classification statistics chart. Blue bars indicate GO terms within the cellular component category. Red bars indicate GO terms related to the molecular function category. Green bars represent GO terms associated with biological processes.

We further conducted a GO enrichment analysis of these 17 genes (Figure 5). However, because only the MaizeGDB database was used as a reference, only 11 genes were ultimately enriched. The results indicated that among the 10 cellular component classifications, the most enriched secondary classifications for these genes were ‘cell’, ‘cell part’, and ‘organelle’, respectively. In the molecular function classification, ‘catalytic activity’ had the highest enrichment proportion among the four secondary classifications, followed by ‘binding’. Among the 13 biological process classifications, ‘cellular process’ was predominant. Notably, the gene *Zm00001d031173* was enriched in both the ‘growth’ and ‘developmental process’ secondary classifications, and both categories correlated with TW.

Based on the functional annotation and GO enrichment analysis of genes, we identified three genes detected in proximity to the co-localized loci with functional annotations or homologous genes, as well as one in the enriched categories related to TW, as candidate genes, including *Zm00001d044362*, *Zm00001d011048*, *Zm00001d011049*, and *Zm00001d031173.*

### 2.6. Genomic Selection Analysis

**Figure 6:**
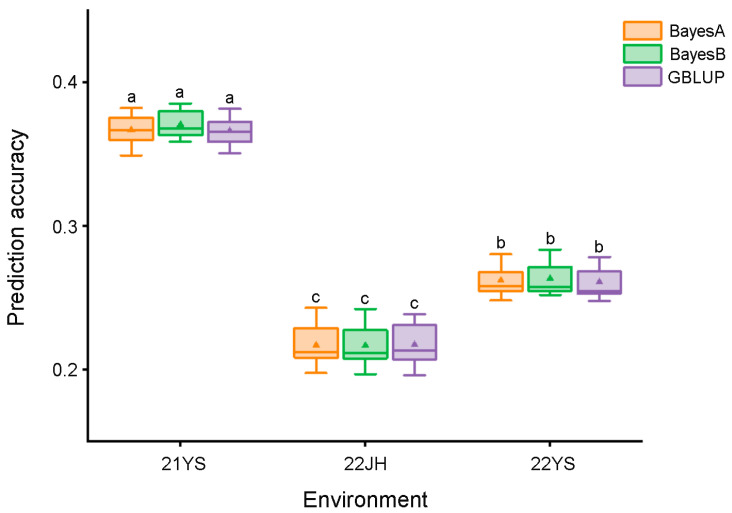
Prediction accuracy of TW in the multi-parent population across different environments using three models. The triangles indicate average prediction accuracy. The letters ‘a’, ‘b’, and ‘c’ denote statistically significant differences between models at the α = 0.05 level in the respective environments.

GS analysis used all 6616 SNPs to model the prediction of maize TW in the multi-parent population across the three environments. Three statistical models were used in this study: BayesA, BayesB, and GBLUP. For each model, 90% of the total population was randomly selected as the training population, with the remaining 10% as the prediction population, and the process was repeated 25 times. The summarized data are shown in Figure 6. In the 21YS environment, the prediction accuracy of each model ranged from 34.90% to 38.19% for the BayesA model, 35.85% to 38.50% for the BayesB model, and 35.06% to 38.13% for the GBLUP. In the 22JH environment, the prediction accuracies for the BayesA, BayesB, and GBLUP models ranged from 19.75% to 24.28%, 19.68% to 24.21%, and 19.60% to 23.84%, respectively. In the 22YS environment, the prediction accuracy of the three models ranged from 24.81% to 28.01%, 25.19% to 28.33%, and 24.79% to 27.84%, respectively. When considering the average prediction accuracy of TW from 25 repetitions, a comparison among the three models revealed that the BayesB model performed best in predicting the tassel weight, achieving a higher prediction accuracy than the other two models. In conclusion, selecting the optimal model and relevant markers can significantly improve the selection efficiency during maize breeding for TW.

## 3. Discussion

### 3.1. Comparison of Significantly Associated Loci for TW with Previously Reported QTLs

We conducted a population structure analysis of 642 F8 RILs within the multi-parent population in our study. The TW phenotypes displayed a normal distribution across the three different environments (Table 1, Figure 2), suggesting likely polygenic control of this trait. Subsequently, a GWAS was performed for TW, which revealed 16 significantly associated SNP loci (Table 2). Notably, two SNPs, S3_226041015 and S8_137379725, consistently exhibited significance in TW across the different environments. In particular, SNP S3_226041015, with the lowest p value, was located within the reported QTL bin 3.09 related to TW [32], indicating that the candidate region of this locus may contain genes that regulate TW. Furthermore, in the 21YS environment, SNP S10_12395326 on chromosome 10 overlapped with the previously reported QTL bin 10.04 governing TW [17]. Although SNP S10_12395326 was detected in only one environment, it suggests the potential presence of genes related to TW within bin 10.04. In contrast, the co-localized SNP S8_137379725, which is not closely linked or overlapped with previously reported TW-related QTLs/genes, falls within the hotspot interval of maize tassel branch number QTLs [33]. This suggests the existence of multiple genes that may regulate tassel-related traits in the vicinity of SNP S8_137379725. The remaining loci, comprising S1_180672691, S1_180672694, S2_158368933, S3_50835736, S3_50835888, S3_50835891, S4_57322369, S4_122010523, S9_51490213, S9_51490247, and S9_51490264, were unique and not closely located or overlapped with the known genes or previously reported QTLs. However, we speculate that loci or genes regulating TW may exist in the vicinity of the regions where these SNPs are clustered on their respective chromosomes (Chr1: 180.672 Mb, Chr3: 50.835 Mb, and Chr9: 51.490 Mb). The disparities observed between our results and previous findings can likely be attributed to the diverse and abundant variation in the tropical maize germplasm used in our population, coupled with the utilization of different environments during the association mapping study. Nevertheless, the consistency of our findings with previously reported results validates the accuracy of our research in identifying these TW-associated loci. The SNPs identified in this study warrant further exploration and provide a crucial theoretical basis for future in-depth studies on maize tassel weight.

### 3.2. Functional Analysis of Candidate Genes

In this study, we conducted a GWAS to map SNPs associated with TW in a multi-parent maize population comprising 642 RILs across three distinct environments. We performed a comprehensive screening of candidate genes within a 250 kb region upstream and downstream of significant SNPs, using MaizeGDB, InterPro, UniProt, and NCBI databases, along with pertinent published research. This comprehensive screening identified 17 genes, with 14 exhibiting functional annotations or homologs (Table 3). Furthermore, 11 of these genes were enriched in the GO analysis. Four genes, namely *Zm00001d044362*, *Zm00001d011048*, *Zm00001d011049*, and *Zm00001d031173*, have emerged as promising candidate genes for TW based on multiple criteria: (1) they are functionally annotated genes located in the vicinity of co-localized SNPs; (2) they are homologous to *Arabidopsis thaliana* genes known for functions related to TW, located near the candidate regions of the co-localized SNPs; and (3) they were enriched in pathways associated with TW during the enrichment analysis.

*Zm00001d044362* encodes Shaggy-related protein kinase alpha (ASKα), a member of the glycogen synthase kinase-3 (GSK-3) subfamily within the CMGC Ser/Thr protein kinase family, representing a unique plant protein kinase. ASKα shares up to 70% sequence similarity with *Drosophila* Shaggy (SGG) and rat GSK-3 [34]. This gene was highly expressed in maize tissues, including the embryo, endosperm, leaves, tassels, and mature pollen. GSK-3 protein kinase, first identified in plants in 1993 [35], has since become a focal point of research. Subsequent investigations of the gene structure, phylogeny, and functional regulation of GSK3/SHAGGY-like kinases have intensified because of their pivotal roles in diverse physiological and developmental processes. These processes encompass cell growth, root and stomatal development, inflorescence development, light responses, and stress responses and are achieved through the phosphorylation of multiple substrates [36,37,38,39]. In *Arabidopsis thaliana*, *BRASSINOSTEROID-INSENSITIVE 2* (*BIN2*) plays a crucial role in steroid signaling regulation. Its mutation or overexpression inhibits brassinosteroid (BR) signal transduction, making *BIN2* a critical negative regulator of plant steroid signaling [40]. This phenomenon has also been observed in maize, where impaired BR biosynthesis leads to the poor development of anthers and pollen, causing male sterility [41]. Similarly, the maize GSK3-like kinase *ZmSK2*, which is homologous to the *Arabidopsis thaliana BIN2* gene, participates in plant embryonic development [42], underscoring the importance of BR signaling in maize tassel and embryonic development. In *Arabidopsis thaliana*, overexpression of the AtSK3-2 mutant reduces cell elongation in the inflorescence [43], high expression of AtSK11 and AtSK12 in the inflorescence, and gene silencing leading to supernumerary perianth, resulting in altered pistil morphology [44]. Moreover, earlier studies have found that *PSK6* [45] in *Petunia hybrida* and *NtK-4* [46] in tobacco are both expressed in mature pollen, further revealing the potential role of GSK3 family protein kinases in the development and function of male inflorescences, including pollen development. The collective research indicates that GSK3 family protein kinases are closely associated with male plant inflorescence development, leading us to postulate that *Zm00001d044362* may indeed play a role in regulating maize TW.

*Zm00001d011048* encodes the pre-mRNA-processing-splicing factor 8A (PRP8A). This gene was highly expressed in mature leaves, embryos, spikelet meristems, and maize tassels. In *Arabidopsis thaliana*, PRP8 has two homologous proteins, PRP8A, also known as SUS2 (*AT1G80070*), and PRP8B (*AT4G38780*). SUS2 is highly conserved in *Arabidopsis*, and mutations in this gene result in abnormal embryonic development and lethality [47]. Furthermore, PRP8A is implicated in the flowering regulation pathway [48]. The PRP8A-6 mutant in *Arabidopsis* influences the expression of *FLOWERING LOCUS C* (*FLC*) by reducing the splicing efficiency of the antisense transcriptional regulator, the *COOLAIR* intron. This alteration leads to H3K4me2 demethylation in the *FLC* coding region, which affects the flowering time. In double mutants of PRP8A and PRP8B, ovules fail to attract pollen tubes, and pollen tubes lose the ability to perceive signals released by the ovules [49]. Additionally, other studies have identified PRP8A as a target gene for alternative splicing [50]. Notably, the PRP8A-14 variant significantly altered the selection of 5′- and 3′- splice sites, displaying a preference for shorter introns. Single and double mutants of PRP8A-14 exhibit physiological and morphological changes including shortened inflorescence stems, reduced fertility, and enlarged leaves. Therefore, we hypothesized that the expression of the candidate gene *Zm00001d011048* plays a crucial role in various plant physiological processes. It could regulate meristem growth, the flowering pathway, and the process of pollen tube attraction, all of which are vital for tassel weight formation.

Although the gene *Zm00001d011049* lacks functional annotation in maize, it displays elevated expression in various parts, including the endosperm, seed, tassel, embryo, root, floret meristem, and spikelet meristem. Its homologous gene in *Arabidopsis* is *AT4G22000*, which is similarly expressed in the flower, pedicel, meristem, embryo, pollen, and stamen. This gene encodes a tyrosine sulfotransferase-like protein (TPST) belonging to the sulfotransferase family (SULT). TPST is instrumental in the catalysis of known plant-sulfated peptide hormones, such as phytosulfokine (PSK), plant peptides containing sulfated tyrosine 1 (PSY1), root meristem growth factor (RGF), Casparian strip integrity factor (CIF1/2), and xylem-associated peptides (XAPs). These substances play crucial roles in signal transduction within plants through sulfation and contribute to various physiological processes [51,52]. For instance, PSK participates in root development [53], pollen germination [54], pollen tube growth and guidance [55], and immune responses [56,57]. PSY1 primarily exhibits high expression in the root elongation zone and apical meristem [58], impacting multiple fungal infections through its signal pathways [56,59]. RGF plays a role in root apical meristem homeostasis [60] and lateral root formation [61]. CIF1/2, acting via the receptor SGN3, is implicated in Casparian strip formation [62]. Through the regulation of Casparian strip membrane domain protein-like (CASPL) and MYB domain protein 15 (MYB15), CIF1/2 induces lignification [63]. Finally, XAPs are involved in lateral root formation [64]. In *Arabidopsis*, *AtTPST* (*TPST-1*) mutants exhibit various developmental anomalies, including stunted roots, pale leaves, fewer higher-order leaf veins, early aging, and shorter inflorescences, characterized by a noticeable reduction in the number of flowers and siliques [65]. Collectively, these findings underscore the significant influence of the TPST family on plant growth and development. Therefore, we deduced that *Zm00001d011049* may be intricately associated with the formation of maize TW, making it a plausible candidate gene.

*Zm00001d031173* encodes the DnaJ protein ERDJ3A, a member of the DnaJ/Hsp40 family. Proteins within this family serve as co-chaperones, interacting with the molecular chaperone heat shock protein Hsp70. They play a crucial role in various processes, including protein folding, transport, assembly, and degradation [66,67,68]. In *Arabidopsis thaliana*, the *Thermosensitive Male Sterile 1* gene (*TMS1*) encodes the J-domain protein AtERdj3A. At 30 °C, a knockout mutant inhibited pollen tube growth in the transmission tract, significantly reducing male fertility [69]. Subsequent research revealed the crucial role of this protein in pollen development and its contribution to plant heat tolerance [70]. Under high-temperature conditions, a mutant of another J-domain protein, AtERdj3B, exhibited lower seed production, a phenomenon effectively alleviated by overexpression of ERDJ3A, which is regulated by the ERDJ3B promoter [71]. In our study, GO enrichment analysis revealed that *Zm00001d031173* is intricately involved in multiple biological processes and molecular functions related to TW. These processes include growth, reproductive development, cell differentiation, pollination, metabolic processes, pollen tube development, apical cell growth, stress responses, cell morphology, and catalytic activity. Based on these findings, our hypothesis posits that the expression of this gene could enhance tassel growth and development, pollen tube development, and apical meristem growth, leading to an increase in maize tassel weight.

This study included functional annotation and enrichment analysis of genes situated upstream and downstream of significant SNPs. Comparative analysis with prior research revealed that the candidate genes identified showed heightened expression levels in maize tassels. Furthermore, the proteins encoded by these genes are intricately associated with pathways governing tassel or meristem development. We identified these four genes as candidates for the regulation of tassel weight. Notably, these TW-related genes are reported for the first time in maize, suggesting that they may represent novel candidate genes regulating TW. Furthermore, the three candidate genes *Zm00001d044362*, *Zm00001d011048*, and *Zm00001d011049* discovered through the two co-localized SNPs have the potential to modulate the expression levels of their corresponding proteins through allelic variations. This modulation can influence the growth and weight of maize tassels. For instance, a C-T substitution in the candidate gene *Zm00001d044362* near SNP S3_226041015 resulted in a significant increase in TW in maize plants with the TT allele. Conversely, a C-T substitution occurring in candidate genes *Zm00001d011048* and *Zm00001d011049* near SNP S8_137379725 led to a slight decrease in TW in maize plants with the TT allele. We also observed that the co-localized SNP S8_137379725 is situated on an LTR-RT located within 30 kb upstream and downstream of the two candidate genes *Zm00001d011048* and *Zm00001d011049* (Figure 4). In maize, the insertion of various transposable elements, such as Hopscotch and solo LTR, has been identified as a mechanism that regulates gene expression, consequently affecting apical dominance [72] and leaf angles [73]. In apple, the insertion of an LTR-RT upstream of *MdMYB1*, a key transcriptional activator controlling anthocyanin biosynthesis, promotes the formation of red skin by influencing the expression of *MdMYB1* [74]. Based on this, we hypothesized that an LTR-RT may similarly influence the expression of candidate genes *Zm00001d011048* and *Zm00001d011049*, thereby exerting control over tassel weight in maize. These findings establish a crucial theoretical foundation for future investigations of the genetic regulatory mechanisms of maize tassels. Furthermore, the significant SNP markers and candidate genes hold promise for potential applications in the efficient selection of maize varieties with the desired tassel weight through marker-assisted selection (MAS) and GS.

### 3.3. Genome-Wide Selection Prediction

Genomic selection has gained widespread application in various crop breeding programs owing to its capacity to expedite breeding cycles and reduce the associated costs [75]. Although there have been few studies on GS analysis of maize tassel-related traits, the rrBLUP model is a commonly employed approach for the analysis of tassel branch number and length. Unlike the rrBLUP model, this study employed BayesA, BayesB, and GBLUP models for the GS of tassel weight. Bayes-type models integrate the variance of the effect values for each marker or chromosome segment as a prior distribution to improve prediction accuracy. We found that the prediction accuracy range for TW was 0.20–0.39, close to the precision for tassel-related traits in previous studies (0.25–0.48) [29,30].

The accuracy and efficiency of GS depend on several factors, including the choice of the prediction model, population size, number of molecular markers, and heritability of the trait [76,77]. In this study, we compared the prediction accuracies of three models under different environmental conditions. Although the BayesB model did not differ significantly from the other two models in the same environment, it exhibited greater stability and higher prediction accuracy across the three different environments. Importantly, our results showed that even with the same model, population, and molecular markers, different environments significantly affected the prediction accuracy (Figure 6), confirming the high susceptibility of maize TW-related traits to environmental factors. Previous studies have shown that the accuracy of trait prediction is closely related to heritability. Therefore, the high environmental sensitivity and low heritability of TW could be the main reasons for its low prediction accuracy. In addition, this study used a limited number of SNP markers. Despite the use of high-quality SNP markers, the lower marker density might not have sufficiently captured the genetic variation associated with TW, thereby affecting the prediction accuracy.

To address these limitations and challenges, we propose that introducing gene–environment interaction effects into the analysis model for traits with lower heritability can enhance prediction accuracy. Regarding molecular marker density, if it is not feasible to significantly increase the number of markers, researchers may consider analyzing widely known trait-related markers as fixed effects in the model. These approaches help enhance the predictive accuracy and efficiency of traits sensitive to the environment and in analyses with low marker density, offering new strategies and directions for future research and practical applications.

## 4. Materials and Methods

### 4.1. Test Materials and Field Experiment

**Figure 7:**
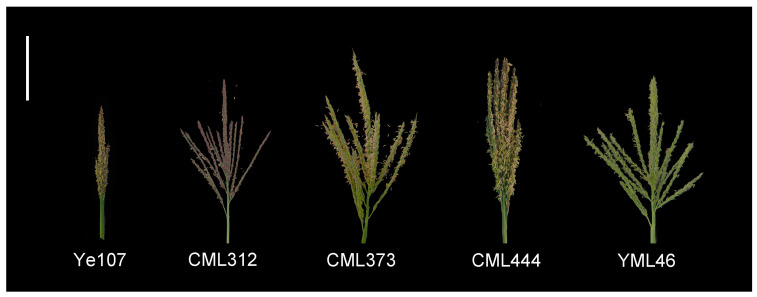
Comparison of tassel size in parental lines of multi-parent population three days after pollination. Bar, 10 cm.

In this study, four tropical maize inbred lines, CML312, CML373, CML444, and YML46, with abundant genetic variation in tassel weight, were selected as female parents and crossed with an elite temperate maize inbred line, Ye107 (male parent), which is widely used in China (Figure 7, Table 4). This resulted in the development of four F1 hybrids. Subsequently, the F1 hybrids were selfed using the single seed descent method for eight generations, ultimately constructing four RIL subpopulations: Pop1 (CML312 × Ye107), Pop2 (CML373 × Ye107), Pop3 (CML444 × Ye107), and Pop4 (YML46 × Ye107), constituting a multi-parent population. Initially, each group contained 200 RILs; however, owing to inbreeding depression, environmental stress, and other factors, only 642 RILs were available for use in this study. These RILs were grown in three environments: Yanshan (YS) in Yunnan Province in 2021 and 2022 and Jinghong (JH) in Yunnan Province in 2022. Field trials were conducted using a completely randomized block design, with two replications in each environment. Each plot consisted of 4 m rows with a row spacing of 70 cm and 14 plants per row. The trials were conducted in accordance with standard agronomic practices.

### 4.2. Phenotypic Data Analysis

Maize tassel weight data were recorded 10 days after anthesis. Starting from the fifth plant in each row, the lowest branch of the tassel and the part above it were collected from five consecutive plants for evaluation. They were air-dried until the weight no longer changed, then weighed, and the average weight was calculated. We utilized IBM SPSS Statistics 26.0 and R software (V4.0.5) for descriptive statistical analysis and variance analysis of the collected phenotypic data. Broad-sense heritability was calculated by using the formula proposed by Knapp et al. [78]:

h2=σg2σg2+σge2e+σε2re×100%

where σg^2^ is the genetic variance, σge^2^ is the genotype–environment interaction variance, σε^2^ is the error variance, e is the number of environments, and r is the number of repetitions.

### 4.3. DNA Extraction and Genotyping-by-Sequencing (GBS)

Genomic DNA was extracted from the seedling leaves of the multi-parent population using the cetyl trimethyl ammonium bromide (CTAB) method [79,80]. After the purity and integrity of the DNA were confirmed, the restriction endonucleases PstI and MspI (New England BioLabs, Ipswich, MA, USA) were used for digestion, and barcode adapters were attached using T4 DNA ligase (New England BioLabs). The DNA library of 300 bp size was constructed and sequenced on the Illumina NovaSeq 6000 platform (Illumina Inc., San Diego, CA, USA) with a read length of 2 × 150 bp. The generated raw sequencing reads were processed to remove reads with adapters and low-quality reads, resulting in high-quality filtered reads. Subsequently, the BWA 0.7.17 was employed to align the filtered reads with the maize reference genome B73_RefGen_v4 [81] using the parameters set at mem -t 4 -k 32 -M. SAMtools 1.9 was used to convert the alignment results into BAM files. SNP calling from the BAM files was carried out using the best practice process of GATK software v4.1.4.0 [82]. SNPs were filtered to select high-quality SNPs using VCFtools software (v0.1.16), with parameters set at --max-missing 0.5 --mac 3 --minQ 30 --minDP 3 --remove lowDP.indv --maf 0.05 --min-meanDP 20 --max-missing 0.95. The filtering process identified 6616 high-quality SNPs for the subsequent GWAS and GS analyses. The identified SNPs were functionally annotated using the ANNOVAR software (v2013-05-20) [83] and the maize reference genome B73_RefGen_v4.

### 4.4. Population Structure Analysis and Genome-Wide Association Study

In this study, principal component analysis (PCA) and kinship analysis were conducted on 642 RILs using the GAPIT package in R software, with the filtered genotypic dataset. To conduct GWAS for tassel weight under three environments (2021YS, 2022YS, and 2022JH), we employed the FarmCPU (fixed and random model circulating probability unification) model using the rMVP package in R software.

Population structure was used as a covariate during the association analysis to reduce the risk of false positives. After association analysis, Manhattan and QQ plots were generated. Applying the Bonferroni correction [84], we set the threshold as −log_10_*p* (where *p* = 1/N, N is the number of SNPs), and we established a threshold of *p* = 1.51 × 10^−4^ to identify the significantly associated SNPs. To identify candidate genes for tassel weight, we mapped significant SNPs to the reference maize genome, B73 RefGen_v4. Considering information from other studies and the relative positions of SNPs, candidate genes were screened 250 kb upstream and downstream of the significant SNPs [85].

### 4.5. Functional Annotation and GO Enrichment Analysis of Candidate Genes

This study initially employed databases such as MaizeGDB, InterPro, UniProt, and NCBI for annotation and functional prediction analysis of candidate genes. Subsequently, the clusterProfiler package (V3.18.1) in R software was used with default parameters to perform GO enrichment analysis of the candidate genes. Hypergeometric testing was used to identify significantly enriched GO terms related to biological processes (BPs), molecular functions (MFs), and cellular components (CCs) in comparison to the genome-wide background.

### 4.6. Genomic Selection

To assess the prediction accuracy of various genomic selection models, we utilized the BGLR package in R for analysis. In this study, we used 6616 SNP markers and implemented three models, namely Bayes A, Bayes B, and Genomic Best Linear Unbiased Prediction (GBLUP), to predict the accuracy of the models for maize tassel weight under three different environments. We implemented a 10-fold cross-validation method by dividing the multi-parent population of 642 RILs into ten equal subsets. During each validation, nine subsets were selected as the training population with both phenotypic and genotypic values, whereas one subset served as the prediction population with only genotypic values for the GS analysis. Each model underwent 25 repeated validations in each environment to ensure result stability.

## 5. Conclusions

In summary, this study conducted a GWAS on tassel weight in a multi-parent maize population, using tropical germplasms as female parents, and identified four novel candidate genes (*Zm00001d044362*, *Zm00001d011048*, *Zm00001d011049*, and *Zm00001d031173*) associated with this trait. By leveraging gene functional annotation and GO enrichment analysis, we propose that these candidate genes may contribute to the growth and development of maize inflorescence with potential involvement in the formation and development of tassel weight. Notably, this study suggests that an LTR-RT can influence the expression of the two candidate genes on chromosome 8. Additionally, our findings highlight that the BayesB model is more accurate in predicting tassel weight during genomic selection. This study provides valuable insights for further research on new candidate genes related to maize tassel weight and a deeper exploration of the genetic regulatory mechanisms governing the traits and morphological structures associated with maize tassels. Additionally, the discovery of candidate genes and genomic prediction for tassel weight offer significant support for future research in terms of validating genes and applying genomic selection to cultivate high-yielding varieties with desirable tassel weight. This will contribute to the improvement of maize tassel morphology and advancements in agricultural practices.

## Figures and Tables

**Table 1:** Descriptive statistical analysis of tassel weight in the multi-parent population.

Population	Environment	Mean	Standard Deviation	Skewness	Kurtosis	Coefficient of Variation	Min–Max	Heritability (%)
Pop1	21YS	1.95	0.67	0.4	0.27	0.34	0.21–4.08	33.10
	22JH	2.06	0.77	0.62	0.34	0.38	0.34–4.44	
	22YS	1.79	0.56	0.37	–0.1	0.31	0.72–3.28	
Pop2	21YS	2.89	1.08	0.38	–0.25	0.37	0.90–5.74	33.00
	22JH	2.74	0.86	0.41	–0.27	0.31	0.90–5.03	
	22YS	2.49	0.9	0.6	0.15	0.36	0.44–5.04	
Pop3	21YS	2.21	0.71	0.22	0.01	0.32	0.70–4.50	33.20
	22JH	2.51	0.91	0.48	0.44	0.36	0.47–5.38	
	22YS	2.27	0.82	0.39	–0.07	0.36	0.60–4.88	
Pop4	21YS	2.05	0.63	0.1	–0.29	0.31	0.62–4.01	33.40
	22JH	2.34	0.79	0.09	–0.48	0.34	0.52–4.14	
	22YS	2.04	0.8	0.66	0.89	0.39	0.48–5.01	

21YS, 22JH, and 22YS represent the trials conducted in Yanshan in 2021, Jinghong in 2022, and Yanshan in 2022, respectively.

**Table 2:** Detection of significant SNPs for tassel weight in the multi-parent population.

Environment	Chromosome	SNP Position	Ref/Alt	*p* Value	Effect
2021Yanshan (21YS)	1	180672691	G/A	6.68 × 10^−5^	0.385
	1	180672694	G/A	2.39 × 10^−5^	0.436
	2	158368933	A/G	9.90 × 10^−5^	0.279
	3	226041015	C/T	8.13 × 10^−6^	−0.440
	9	51490213	G/T	1.11 × 10^−4^	0.274
	9	51490247	G/A	7.68 × 10^−5^	0.278
	9	51490264	G/T	1.06 × 10^−4^	0.274
	10	12395326	T/C	3.37 × 10^−5^	1.934
2022Jinghong (22JH)	3	226041015	C/T	1.28 × 10^−4^	−0.398
	8	137379725	C/T	1.29 × 10^−4^	0.372
2022Yanshan (22YS)	3	50835736	T/C	1.08 × 10^−4^	0.329
	3	50835888	G/A	1.42 × 10^−4^	0.341
	3	50835891	A/T	1.42 × 10^−4^	0.341
	4	57322369	A/T	1.48 × 10^−4^	1.234
	4	122010523	A/T	2.33 × 10^−5^	0.265
	8	137379725	C/T	4.88 × 10^−5^	0.368

**Table 3:** Significant SNPs associated with tassel weight in the multi-parent population.

SNP	Gene ID	Functional Annotation
S1_180672691	Zm00001d031171	Cactin
S1_180672694	Zm00001d031173	DnaJ protein ERDJ3A
S2_158368933	Zm00001d005102	Protein TONNEAU 1b
	Zm00001d005103	—
S3_50835736	Zm00001d040573	B3 domain-containing transcription repressor VAL2
S3_50835888	Zm00001d040577	—
S3_50835891		
S3_226041015	Zm00001d044359	—
	Zm00001d044362	Shaggy-related protein kinase alpha
S4_57322369	Zm00001d049986	Pentatricopeptide repeat-containing protein mitochondrial
	Zm00001d049987	Probable potassium transporter 4
S4_122010523	Zm00001d050771	Tetratricopeptide repeat (TPR)-like superfamily protein
	Zm00001d050774	CCR-like
S8_137379725	Zm00001d011048	pre-mRNA-processing-splicing factor 8A
	Zm00001d011049	—
S9_51490213	Zm00001d045978	Protein MICRORCHIDIA 6
S9_51490247		
S9_51490264		
S10_12395326	Zm00001d023619	Mono-/di-acylglycerol lipase N-terminal; Lipase class 3
	Zm00001d023620	—

**Table 4:** Parental lines used for multi-parent population development.

Parent	Pedigree	Heterotic Group	Ecological Type
Ye107	Selected from US hybrid DeKalb XL80	Reid	Temperate
CML312	S89500-F2-2-2-1-1-B	Non-Reid	Tropical
CML373	P43SR-4-1-1-2-1-B-8-1-B	Non-Reid	Tropical
CML444	P43C9-1-1-1-1-1-BBBB	Non-Reid	Tropical
YML46	SW1-1-1-2-1-2-1	Suwan	Tropical

## Data Availability

The data presented in this study will be available on request from the corresponding author.
